# Efficacy and safety of low dose aspirin plus clopidogrel in the treatment of elderly patients with symptomatic intracranial artery stenosis

**DOI:** 10.3389/fneur.2023.1060733

**Published:** 2023-03-02

**Authors:** Hai-xia Song, Bin Zhang, Shan Liu, Zhi-chao Shi, Zi-yun Wang, Hai-li Lu, Jie Yao, Juan Chen

**Affiliations:** Department of Neurology, Shijiazhuang People's Hospital, Shijiazhuang, Hebei, China

**Keywords:** low dose DAPT, elderly, symptomatic intracranial artery stenosis, efficacy, safety

## Abstract

**Background:**

As one of the most common causes of stroke, symptomatic intracranial artery stenosis (sICAS) is a great threat to public health, and its financial burden is substantial, with annual direct high medical costs particularly in China. Currently, the long-term use of conventional dual antiplatelet therapy (DAPT) as the primary modality of treatment for sICAS decreases the risk of stroke recurrence but increases the risk of bleeding. We aimed to evaluate the efficacy and safety of low dose aspirin plus clopidogrel for the treatment of sICAS in the elderly population.

**Methods:**

This randomized, controlled study included 181 older patients with transient ischemic attack (TIA) or ischemic stroke (IS) attributed to sICAS, who were recruited between April 2015 and November 2020. The 90 patients assigned to the low dose therapy group included aspirin, 75 mg, plus clopidogrel, 50 mg, daily for 90 and 91 patients assigned to the conventional group included aspirin, 100 mg, plus clopidogrel, 75 mg, daily for 90 days (aspirin or clopidogrel alone daily thereafter) were included in this intention-to-treat analysis. Efficacy and safety analyses were done in this trial.

**Results:**

One hundred eighty-one eligible elderly patients with sICAS were enrolled in this trial. The median age was 70 years ranged 60–83 years. Seventy-five participants were with TIA and 106 with IS. The median time of follow-up was 30 months ranged 1–36 months. Ninety patients were assigned randomly to the low dose group and 91 patients to the conventional group. The rate of primary, secondary and composite efficacy were not significantly different between the low dose and conventional group (*P* > 0.05). The rate of composite safety outcome was 7.8% (7/90) in the low dose group, which was lower than 17.6% (16/91) in the conventional group (χ^2^ = 3.921, *P* = 0.048). At the time of last follow-up, 17 (9.4%) of 181 patients developed GI injuries, which occurred in four (4.4%) of 90 patients in the low dose group and in 13 (14.3%) of 91 patients in the conventional group (χ^2^ = 4.058, *P* = 0.044). The primary efficacy outcome occurred in six (18.2%) of 33 patients with severe sICAS and in 22 (38.6%) of 57 patients with moderate sICAS (χ^2^ = 4.064, *P* = 0.044) in the low dose group.

**Conclusion:**

In this study, the safety of low dose aspirin combined with clopidogrel proved to be equally efficient and significantly safer than those of conventional dose within 24 months in elderly patients with sICAS. However, the small size of this study limits the validity of the results. Further larger longitudinal and randomized controlled trials are necessary to evaluate the role of low dose DAPT in the patients with sICAS.

## Introduction

As one of most common causes of stroke, intracranial atherosclerotic stenosis (ICAS) is prevalent among the Asians, especially in China (1). The data from the Chinese study about transient ischemic attack (TIA) and ischemic (IS) caused by symptomatic (sICAS) with a high risk recurrence indicated that the prevalence is as high as 30−46% (2, 3). Actually, ICAS is also an independent risk factor for IS in Asian and African-American patients, with high stroke recurrence rate for some pathological reasons (4). The therapeutic methods of sICAS nowadays primarily include medical management and interventional therapy with control of risk factors (5).

The mechanism of action of DAPT as medical treatment included reducing the synthesis of thromboxane A2, blocking ADP binding and inhibition of P2Y12 receptors. Through these mechanisms, antiplatelet medical therapy can prevent TIA or IS. As an immediate resuscitative therapy for sICAS, percutaneous transluminal angioplasty and stenting (PTAS) is mainly applicable to some vascular lesions with distal ischemia caused by arterial stenosis (6), with the aim to achieve the revascularization of target lesion (7). Additionally, this technique is used extensively in the revascularization of coronary artery, lower extremity artery (8, 9). PTAS is considered to modify stenosis, improve hemodynamics and further reduce stroke recurrence, but its efficacy and safety remains controversial in patients with sICAS (10). The Stenting and Aggressive Medical Management for Preventing Recurrent Stroke in Intracranial Stenosis (SAMMPRIS) (11) and The China Angioplasty and Stenting for Symptomatic Intracranial Severe Stenosis (CASSISS) (12) provided an updated strategy that medical treatement is still the main therapeutic strategy for secondary prevention for sICAS, and do not support the addition of PTAS to medical therapy.

Moreover, despite medical therapy reducing stroke recurrence in sICAS, some patients still face a high bleeding rate. At present, there are few study on the analysis of different antiplatelet doses to evaluate the efficacy and safety in elderly patients with sICAS (10), and many researchers have attempted to improve current DAPT dose to improve safety. Based on this premise, this study aimed to evaluate the low-dose DAPT might enhance the preventive effects of conventional DAPT and address the prevalence with the risk of increasing bleeding of conventional DAPT therapy among Chinese population with sICAS.

## Materials and methods

### Study participants

The population of this retrospective randomiazed study was enrolled in the people's hospital in Shijiazhuang, China from April 2015 to November 2020. All patients were diagnosed with sICAS measured according to the North American Carotid Endarterectomy Test criteria (13). Inclusion and exclusion criteria for the participant in the study were listed in Table 1.

**Table 1:** Inclusion and exclusion criteria.

Inclusion criteria	[1] Age ≥60 years [2] Stenosis of a major intracranial artery supplying the territory of the ischemic event ≥50% by Computed Tomography Angiography (CTA) [3] IS identified on MRI or TIA within the last 3 weeks [4] Iinformed consent from patients or their legal representatives
Exclusion criteria	[1] Contraindication or allergy to antiplatelet agents or contrast [2] Uncontrolled severe hypertension: systolic blood pressure ≥180 mmHg or diastolic blood pressure ≥115 mmHg [3] Severe vascular diseases such as intracranial aneurysm, moyamoya disease, etc. [4] Cardioembolic IS or TIA, and high-risk sources of cardio-embolism [5] History of symptomatic non-traumatic intracranial hemorrhage [6] Use of anticoagulants and non-steroidal anti-inflammatory drugs [7] Any other haemorrhagic disease [8] Bleeding predisposition [9] Blood clotting disorders

Finally, a total of 181 patients diagnosed with sICAS were recruited to this study. The protocolwas reviewed and approved by the ethics committee of Shijiazhuang people's hospital (20211280). All patients or their legal representatives provided their written informed consent to participate in this study, and changes in medications were not permitted after informed consent was obtained.

### Treatment

All patients were randomly assigned to the low dose group (aspirin (Bayer Healthcare Ltd.) 75 mg/day and clopidogrel (Shenzhen Xinlitai Pharmaceutical Co., Ltd.) 50 mg/day) and the conventional group (aspirin 100 mg/day and clopidogrel 75 mg/day) in a 1:1 ratio assigned by a random number table, and all patients were masked to the treatment allocation. The protocol treatment of DAPT was started after expeditious evaluation and definitive diagnosis of sICAS, for a duration of 90 days from the latest ischemic symptom onset. After that, patients were taking monotherapy antiplatelet therapy. To reduce the risk of recurrent stroke and vascular events, clinicians recommend patients enrolled in this study that high-intensity statin therapy to achieve a goal LDL < 70 mg/dl and a long-term BP target of < 140/90 mmHg in clinically stable patients. Aggressive medical management also comprises life-style management that incorporates exercise, smoking cessation and weight management.

### Follow-up

Patients had to be able to visit the study department throughout the follow-up period. The visits were conducted by a combination of outpatient and telephone review. The patients all underwent CTA, transcranial Doppler ultrasound (TCD), and carotid ultrasound. The efficacy and safety outcomes were always evaluated by the physicians who were masked to treatment allocation. Follow-up intervals were at 30 days, 3 months, 6 months, and every 6 months thereafter. Patients who stopped taking the trial drugs for longer than 2 weeks were withdrawn from the trial. Participants who did not develop any event or were lost to follow-up at the last observational date were treated as censored.

### Definitions and measurement

According to World Health Organization criteria, TIA was defined as acute onset of neurologic deficit, persisting for < 24 h. IS was defined as persisting >24 h, confirmed by diffusion-weighted imaging on MRI. According to American Academy of Neurology, sICAS is defined as TIA or ischemic stroke attributed to 50%−99% atherosclerotic stenosis of a major intracranial artery (14). Moderate sICAS was defined as stenosis of 50%−69% and severe ICAS was defined as stenosis of 70%−99%. A major intracranial artery was defined as the level of A2 (the postcommunicating segment of the anterior cerebral artery), M2 (the Sylvian segment of the middle cerebral artery) or P2 (the ambient segment of the posterior cerebral artery) (15). The primary efficacy outcome was defined as the first recurrence of TIA or IS. The secondary efficacy outcome was defined as hemorrhagic stroke, death from any cause, and other vascular events including myocardial infarction, pulmonary embolism, heart failure, aortic dissection and peripheral artery disease requiring hospital admission. The Composite efficacy outcome included both primary and secondary efficacy outcome. The primary safety outcome was defined as major bleeding resulting in substantial haemodynamic instability requiring hospitalization and/or blood transfusion. The secondary safety outcome was defined as gastrointestinal (GI) injury excluded major bleeding, and minor bleeding included skin mucosa gingival bleeding. GI injury was defined as dyspepsia, gastroduodenal ulcers, perforation and bleeding. The Composite safety outcome included both primary and secondary safety outcome. Coronary artery disease was defined history of angina, myocardial infarction, or prior coronary artery revascularization (9). Any event related to the outcomes was reviewed by professionals who were masked to DAPT medications. All workers who were provided access to the results were asked to sign a confidentiality agreement to ensure that the results were not disclosed before presentation of the results.

### Statistical analysis

All data provided in the statistical analysis plan were analyzed using SPSS 26.0 software (SPSS, Inc., Chicago, USA). Results are summarized as means ± standard deviation and range for continuous variables and, and categorical data are summarized as counts or percentages. The data was analyzed using χ^2^ test for categoric variables and the *t*-test or *F*-test for continuous variables. All data were tested for normality using the K–S test. If data were not normally distributed, a M–W *U*-test was applied. The Kaplan–Meier method was used to calculate estimated cumulative event rates, and the treatment groups are compared using the log-rank test. A *P-*value of < 0.05 was considered significant.

## Results analysis

### Baseline characteristics

One hundred eighty-one eligible patients with sICAS, between 12 days and 61 days before the start of the protocol treatment were enrolled. The median age was 70 years (60–83). Ninety patients were assigned randomly to the low dose group and 91 patients to the conventional group. Risk factors included current smoking, hypertension, diabetes mellitus, hyperlipidemia, chronic kidney disease, peripheral arterial disease, history of ischaemic stroke, and coronary heart disease. There was no significant difference in clinical baseline characteristics between the conventional group and the low dose group (*P* > 0.05). Characteristics at baseline are shown in Table 2.

**Table 2:** Baseline characteristics of study participants (*n*, %).

	**Low-dose (*n* = 90)**	**Conventional (*n* = 91)**	**χ^2^/*t***	***P*-value**
**Sex (** ***n*** **, %)**			1.270	0.60
Male	53 (58.9)	46 (50.5)		
Female	37 (41.1)	45 (49.5)		
Asian ethnicity^#^	90 (100.0)	91 (100.0)	–	–
Age (year, mean ± SD)	71.5 ± 6.6	69.8 ± 6.6	1.741	0.083
BMI (kg^2^/m, mean ± SD)	26.8 ± 4.2	27.5 ± 4.4	−1.030	0.304
Current smoking (*n*, %)	48 (53.3)	45 (49.5)	0.273	0.601
**Cerebrovascular event (** ***n*** **, %)**			0.479	0.489
TIA	35 (38.9)	40 (44.0)		
IS	55 (61.1)	51 (56.0)		
Hyperlipidemia (*n*, %)	55 (61.1)	45 (49.5)	2.488	0.115
Hypertension (*n*, %)	63 (70.0)	65 (71.4)	0.045	0.833
Diabetes (*n*, %)	11 (12.2)	12 (13.2)	0.038	0.846
Chronic kidney disease (*n*, %)	6 (6.7)	5 (5.5)	0.109	0.741
Coronary artery disease (*n*, %)	8 (8.9)	11 (12.1)	0.493	0.483
History of ischaemic stroke^*^ (*n*, %)	5 (5.6)	3 (3.3)	0.143	0.706
Peripheral arterial disease (*n*, %)	6 (6.7)	5 (5.5)	0.109	0.741
Stenosis (%, mean ± SD)	74.0 ± 14.7	77.6 ± 15.9	−1.570	0.116
**Degree of stenosis (** ***n*** **, %)**			0.134	0.714
Severe	57 (48.9)	60 (66.7)		
Moderate	33 (36.7)	31 (34.1)		
Time to randomization after latest ischemic event (days)	33.7 ± 14.1	35.1 ± 14.2	−0.676	0.500

^#^Self-reported; all are reported as Chinese.

^*^Except the qualifying stroke for this trial.

The median time of follow-up was 30 months (1–36). At the last time, follow-up data were available for 171 patients. Ten cases were lost during follow-up, included 6 cases in the low dose group and four cases in the conventional group. Median time to randomization after index events was 35.5 days (12–59) in low dose group and 33.0 days (12–61) in conventional group.

### Efficacy and safety outcomes

**Figure 1:**
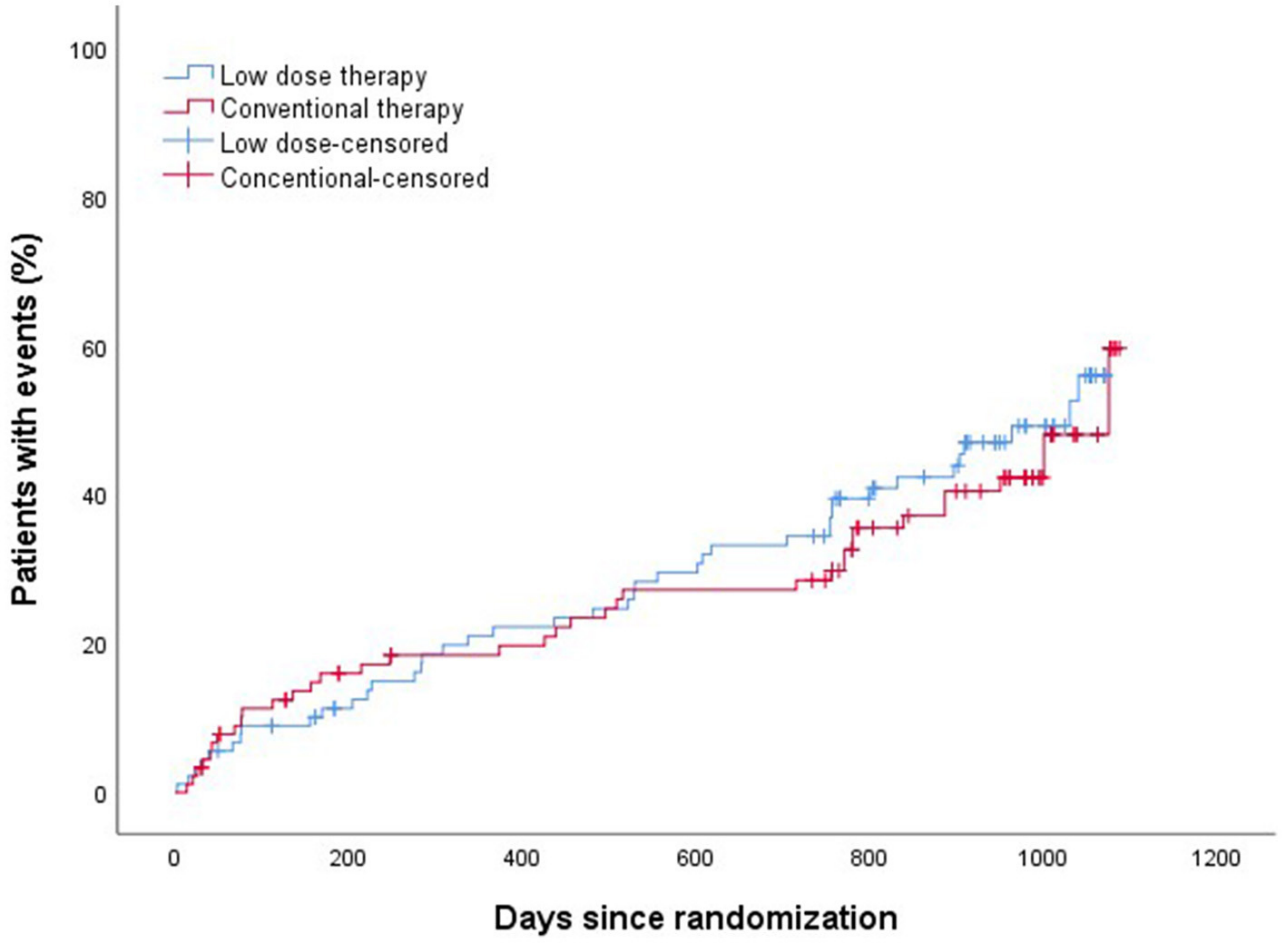
Kalpan–Meier analysis of composite efficacy outcomes.

**Figure 2:**
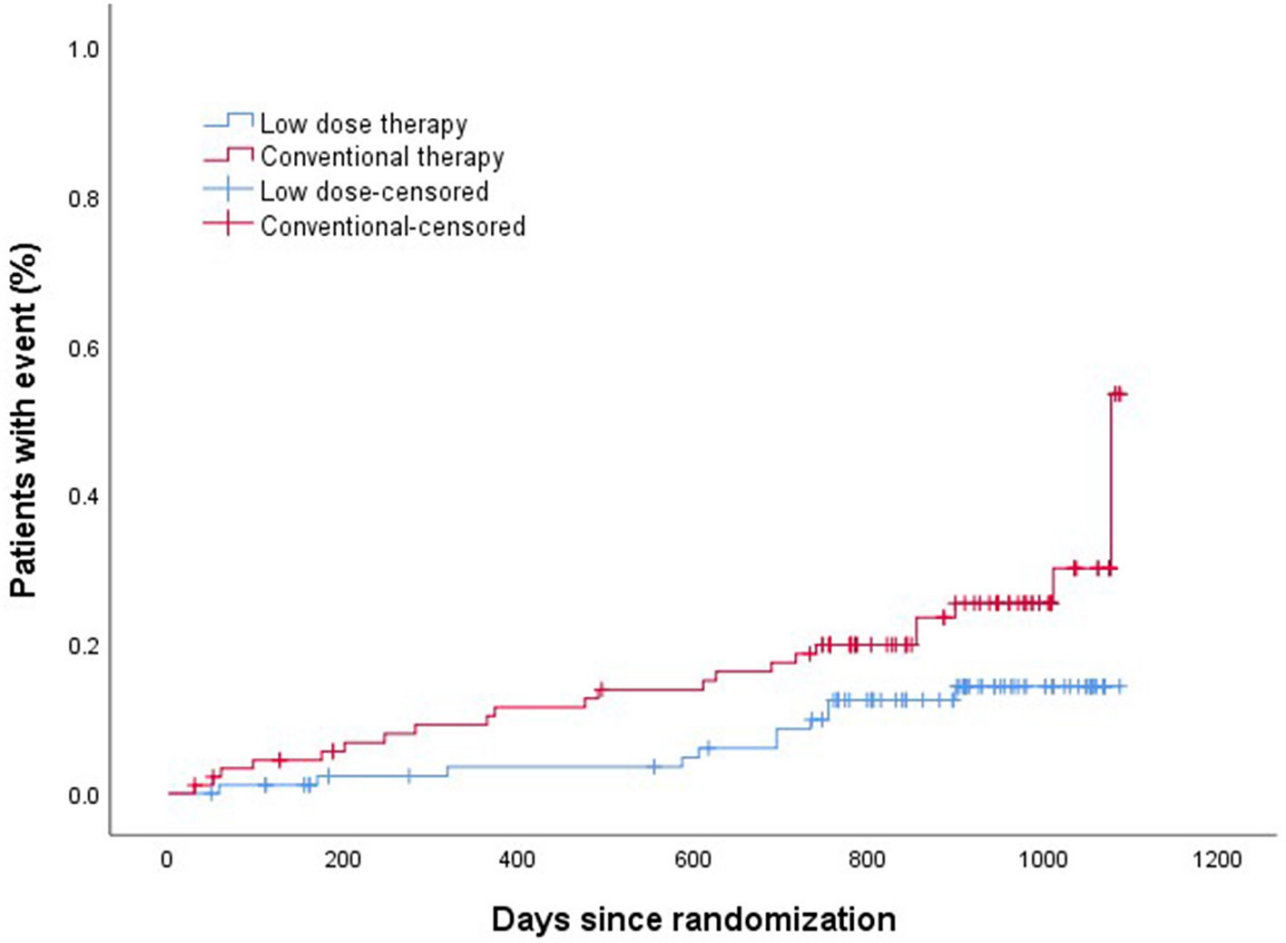
Kalpan–Meier analysis of composite safety outcomes.

The efficacy and safety outcomes a at the 24 months of follow up were shown in Table 2. The efficacy and safety outcomes in the low dose group compared with the conventional group at the 24 months were shown in Table 3 and Figures 1, 2. The rate of primary, secondary and composite efficacy were not significantly different between the low dose and conventional group (*P* > 0.05). The rate of composite safety outcome was 7.8% (7/90) in the low dose group, which was lower than 17.6% (16/91) in the conventional group (χ^2^ = 3.921, *P* = 0.048).

**Table 3:** Efficacy and safety outcomes in the low dose group and conventional group (*n*, %).

	**Low dose group (*****n*** = **90)**				**Conventional group (*****n*** = **91)**			
	**30 days**	**3 months**	**12 months**	**24 months**	**30 days**	**3 months**	**12 months**	**24 months**
**Efficacy**								
Primary outcome	3 (3.3)	6 (6.7)	12 (13.3)	20 (22.2)	2 (2.2)	7 (7.7)	13 (14.3)	19 (20.9)
Secondary outcome	1 (1.1)	2 (2.2)	6 (6.7)	12 (13.3)	1 (1.1)	3 (3.3)	4 (4.4)	8 (8.8)
Composite outcome	4 (4.4)	8 (8.9)	18 (20.0)	29 (32.2)	3 (3.3)	10 (11.1)	16 (17.6)	24 (26.4)
**Safety**								
Primary outcome	0 (0.0)	0 (0.0)	1 (1.1)	1 (1.1)	0 (0.0)	1 (1.1)	1 (1.1)	3 (3.3)
Secondary outcome	0 (0.0)	0 (0.0)	3 (3.3)	7 (7.8)	1 (1.1)	2 (2.2)	7 (7.7)	13 (14.3)
Composite outcome	0 (0.0)	0 (0.0)	3 (3.3)	7 (7.7)	1 (1.1)	3 (3.3)	8 (8.8)	16 (17.6)

### Evaluation of gastrointestinal injuries

At the time of last follow-up, 17 (9.4%) of 181 patients developed GI injuries, which occurred in four (4.4%) of 90 patients in the low dose group and in 13 (14.3%) of 91 patients in the conventional group (χ^2^ = 4.058, *P* = 0.044). Major bleeding occurred in 1 (1.1%) of 90 patients than in the low dose group and in three (3.3%) of 91 patients in the conventional group, all of them recovered from their illness. In addition to this, two (2.2%) cases of minor bleeding and one (1.1%) case of gastric ulcer occurred in the low dose group (Table 4). Six cases of minor bleeding, two (2.2%) cases of gastric ulcer, one (1.1%) case of gastric perforation because of ulcer, one (1.1%) case of dyspepsia and one (1.1%) case of abdominal pain occurred in the conventional group.

**Table 4:** Efficacy and safety outcomes of comparison low dose and conventional at 24 months (*n*, %).

	**Low-dose group (*n* = 90)**	**Conventional group (*n* = 91)**	**χ^2^**	***P***
**Efficacy**				
Primary outcome	20 (22.2)	19 (20.9)	0.048	0.826
Secondary outcome	12 (13.3)	8 (8.8)	0.950	0.330
Composite outcome	29 (32.2)	24 (26.4)	0.747	0.387
**Safety**				
Primary outcome	1 (1.1)	3 (3.3)	0.244	0.621
Secondary outcome	7 (7.8)	13 (14.3)	1.950	0.163
Composite outcome	7 (7.8)	16 (17.6)	3.921	0.048

### Efficacy and safety outcomes of sICAS with different stenosis

At the time of last follow-up, in the low dose group, the primary efficacy outcome occurred in six (18.2%) of 33 patients with severe sICAS and in 22 (38.6%) of 57 patients with moderate sICAS (χ^2^ = 4.064, *P* = 0.044). The rate of secondary and composite efficacy outcomes were higher in severe sICAS group (*P* > 0.05). In the conventional group, the rate of primary, secondary and composite efficacy outcomes were higher in severe sICAS group (*P* > 0.05). The rate of composite safety outcome with severe sICAS was higher in the low dose group and lower in the conventional group (*P* > 0.05; Table 5).

**Table 5:** Efficacy and safety outcomes of sICAS with moderate or severe stenosis (*n*, %).

	**Low dose group (*****n*** = **90)**				**Conventional group (*****n*** = **91)**			
	**Moderate (*****n*** = **33)**	**Severe (*****n*** = **57)**	χ^2^	***P***	**Moderate (*****n*** = **31)**	**Severe (*****n*** = **60)**	χ^2^	***P***
**Efficacy**								
Primary outcome	6 (18.2)	22 (38.6)	4.064	0.044	8 (25.8)	19 (31.7)	0.336	0.562
Secondary outcome	6 (18.2)	11 (19.3)	0.017	0.896	5 (16.1)	10 (16.7)	0.004	0.948
Composite outcome	12 (36.4)	29 (50.9)	1.755	0.183	11 (35.5)	26 (43.3)	0.522	0.470
**Safety**								
Primary outcome	0 (0.0)	1 (3.0)	0.000	1.000	2 (2.2)	1 (1.1)	0.351	0.554
Secondary outcome	1 (3.0)	10 (17.5)	2.862	0.091	8 (8.8)	12 (13.2)	0.402	0.526
Composite outcome	1 (3.0)	10 (17.5)	2.862	0.091	10 (32.5)	13 (21.7)	1.214	0.271

## Discussion

The treatment of sICASis a hot issue of clinical research, but the choice between medical therapy and PTAS has been controversial. Studies found that PTAS can partially restore neurological function and reduce mortality in patients with sICAS, but complications related to ischemia-reperfusion, embolic dislodgement, and bleeding can increase recurrence and mortality, seriously affecting the clinical outcomes (16). The CASSISS trial (12) concluded that the addition of PTAS to medical therapy, compared with medical therapy alone, resulted in no significant difference in the risk of stroke or death within 30 days among patients with TIA or IS due to sICAS. To date, studies have demonstrated the benefit of DAPT for primary and secondary prevention of TIA or IS, and for patients with sICAS, it is recommended that DAPT should be applied in early stage and long-term after index events (5). Given the comparable benefit of aspirin in the range of 50–325 mg/day for the prevention of IS (17), Liu et al. (18) found the greatest benefit of DAPT for 90 days in patients with sICAS. In combination with the advanced age of patients, this single-center observational study was to evaluate the efficacy and safety outcomes of low dose DAPT for the treatment of elderly patients with sICAS. Our aim was to test the efficacy and safety of the low dose DPAT treatment in a controlled analysis of patients with sICAS, and this study shown that the recurrence of TIA or IS was similar with the low dose therapy than conventional dose, and safety outcome of the low dose group are better than conventional group. In this study of secondary stroke prevention in patients with sICAS, we found no evidence of a higher risk of recurrence of adverse events with low dose than conventional therapy. The incidence of the efficacy outcomes in the low dose and conventional group was almost same, and the reoccurrence rates of TIA or IS at 12 and 24 months were 13.3% (12/90) and 22.2% (20/90) in the low dose group and 14.3% (13/91) and 20.9% (19/91) in the conventional group, respectively, both of which and other efficacy outcomes were higher than those previously reported (15, 19). This outcome can be attributed to the following: first, all patients in this trial were elderly. Persoon et al. (20) reported that age was a risk factor for the first or recurrence of sICAS, which was considered that the blood vessels of elderly patients become both sclerotic and stenotic. Second, the patients with TIA were selected for the present study that increased the rate of adverse event rates. Finally, conventional DAPT could prevent stenosis progression of patients with sICAS, and it may have no this effect by the low dose DAPT in this study, although the change in arterial stenosis during the follow-up period was not assessed in this trial. Bleeding from DAPT use most frequently occurred in the digestive system (21). Long-term use the antiplatelet therapy and aging itself can increase risk of GI bleeding (22). It is know that GI bleeding and ulceration from DAPT use increase in severity and frequency with increasing age, and NSAID use as exclusion criteria in this trial increases the risk of GI bleeding in the elderly four folds (23). In this analysis, the incidence of bleeding as a core safety outcome rate in low dose group was significantly lower than that in the conventional group, although their low incidences might weaken statistical power. Another common adverse event of DAPT in elderly patients isGI injury. The incidence of GI injury in this study was 14.3% (13/91) in the conventional group compared to4.4 (4/90) in the low dose group (*P* < 0.05), which may be associated with increased GI toxicity due to high dosage of DAPT. Tegos et al. (24) and Kasner et al. (25) reported a linear positive correlation between the severity of ICAS and the incidence of stroke. Our result was highly consistent with the findings in above mentioned studies.

The evolution of other medical treatment has advanced improving the efficacy and safety outcomes of sICAS. Toyoda et al. (15) reported that the combination of cilostazol with aspirin or clopidogrel had a reduced incidence of IS recurrence and a similar risk of severe or life-threatening bleeding. Amarenco et al. (26) concluded that ticagrelor was superior to aspirin at preventing stroke in a prespecified exploratory analysis. These findings provided effective therapeutic options to prevent adverse events. However, guidelines do not prove the final of submitted version of taking these drug until now.

Patients with sICAS are often comorbid with risk factors correlated with stroke recurrence, including hypertension, metabolic disorders, dyslipidaemia (4). Our study also support the management of vascular risk factors to reduce the risk of recurrent stroke and vascular events. Evidence for the use of high-intensity statins is applicable to patients with sICAS (27, 28). Analyses form Chinese Intracranial Atherosclerosis have demonstrated that a mean systolic BP < 140 mm Hg was associated with a good follow-up outcome, among clinically stable patients with sICAS (29).

Despite the emerging research results, there still persist lots of problems and concerns in clinical practice. For example, this medical therapy for secondary prevention of sICAS in this study is mainly concentrated on population of urban areas. To better balance medical resources, the population of countryside patients should be included in this study. However, the majority of this patients with are TIA or IS has poor compliance with the DAPT secondary prevention (30), and neurological care services need to be enhanced at the country-level hospitals to improve health care delivery.

## Study limitations

There are several limitations in this study. First, this was a retrospective observational study involving a small cohort and the risk of all sorts of bias and confounding is substantial, which limited external validity of any finding or conclusion. Multivariate analysis could not be done to the relatively small size in a single center. Second, the trial treatment was not masked. Thus, the trial investigators were aware of the study drug allocation. Third, long-term of follow-up data which is important indicator of therapy success are not available. Finally, generalizability of the present finding to other countries is uncertain because the ethnicity was limited to Chinese people within this research.

## Conclusions

This study represents a conceptual advance over previously published work. The low dose of DAPT involving aspirin plus clopidogrel was similar effective but safer than the conventional dose during non-cardioembolic IS or TIA with sICAS. The results of the present study support the use of the low dose of DAPT in elderly patients with sICAS. In addition, the study found that the recurrence rate of IS or TIA in patients with severe symptoms of ICAS is higher than that in elderly patients with moderate symptoms of ICAS in the two groups during fellow-up. Further studies should be performed to evaluate the efficacy and safety of low dose DAPT in a long-term follow-up.

## Data availability statement

The raw data supporting the conclusions of this article will be made available by the authors, without undue reservation.

## Ethics statement

The studies involving human participants were reviewed and approved by Shijiazhuang People Hospital of the Ethics Committee (No. 2021031). The patients/participants provided their written informed consent to participate in this study. Written informed consent was obtained from the individual(s) for the publication of any potentially identifiable images or data included in this article.

## Author contributions

Z-yW and Z-cS participated in the design of this study. H-xS drafted and wrote the main manuscript text. BZ and SL collected clinical and demographic data. JY and JC performed the statistical analysis. H-lL prepared Tables 1–5 and Figures 1, 2. All authors critically reviewed the manuscript and agreed on this final version to be submitted to the journal.
